# Targeting PCSK9 Ameliorates Graft Vascular Disease in Mice by Inhibiting NLRP3 Inflammasome Activation in Vascular Smooth Muscle Cells

**DOI:** 10.3389/fimmu.2022.894789

**Published:** 2022-05-26

**Authors:** Yanqiang Zou, Zhang Chen, Xi Zhang, Jizhang Yu, Heng Xu, Jikai Cui, Yuan Li, Yuqing Niu, Cheng Zhou, Jiahong Xia, Jie Wu

**Affiliations:** Department of Cardiovascular Surgery, Union Hospital, Tongji Medical College, Huazhong University of Science and Technology, Wuhan, China

**Keywords:** proprotein convertase subtilisin/kexin type 9, graft vascular disease, NLRP3, vascular smooth muscle cells, transplantation

## Abstract

**Background:**

Graft vascular disease (GVD), which limits the long-term survival of patients after solid-organ transplantation, is associated with both immune responses and nonimmune factors, including dyslipidemia. Recent studies have shown that inhibition of proprotein convertase subtilisin/kexin type 9 (PCSK9), a U.S. Federal Drug Administration-approved treatment for hyperlipidemia, reduces cardiovascular events, regulates inflammatory responses, and enhances the efficacy of immune checkpoint therapy in cancer treatment through a cholesterol-independent mechanism. However, whether targeting PCSK9 is a potential therapeutic strategy for GVD remains unknown.

**Methods:**

Serum samples and grafts were harvested from male mice undergoing abdominal aortic transplantation. The pathological alterations in the aortic grafts were detected by hematoxylin and eosin staining, Verhoeff’s Van Gieson staining, and Masson staining. Inflammatory cell infiltration and proinflammatory cytokine expression in the aortic grafts were detected by immunohistochemistry and quantitative real-time polymerase chain reaction (qRT-PCR), respectively. The regulatory effects of PCSK9 on vascular smooth muscle cell (VSMC) migration and proliferation were examined by transwell, EdU, and western blot assays. The effect of Evolocumab, a PCSK9 inhibitor, on GVD in humanized PCSK9 mice was also evaluated.

**Results:**

PCSK9 was upregulated in the serum, grafts, and liver of mice in the allograft group subjected to abdominal aortic transplantation. *Pcsk9* knockout significantly reduced vascular stenosis, the intimal hyperplasia area and collagen deposition. *Pcsk9* depletion also inhibited macrophage recruitment and the mRNA expression of proinflammatory cytokines in aortic grafts. Furthermore, *Pcsk9* knockout suppressed the migration and proliferation of VSMCs, which was related to the inhibition of NLRP3 inflammasome activation. Meanwhile, Evolocumab significantly ameliorated GVD in humanized PCSK9 mice.

**Conclusion:**

PCSK9 is upregulated in a mouse model of GVD, and *Pcsk9* knockout reduces vascular occlusion, suggesting that PCSK9 may be a promising target for the treatment of GVD.

## Background

With the advances in surgical techniques for organ transplantation and the application of immunosuppressive drugs (tacrolimus, cyclosporine, mycophenolate mofetil, azathioprine, everolimus, sirolimus, etc.), the short-term survival of solid-organ transplantation patients has been greatly improved (1). However, the long-term survival of transplantation patients is still limited. Graft vascular disease (GVD) is a major problem limiting the long-term survival of solid-organ transplantation patients (2). According to the thirty-sixth adult heart transplantation report-2019, the cumulative experienced morbidity rates of cardiac allograft vasculopathy were 8%, 29%, and 47% at 1, 5, and 10 years after heart transplantation, respectively (3). The main pathological features of GVD are vascular intimal hyperplastic lesions composed of smooth muscle-like cells and extracellular matrix, as well as inflammatory cell infiltration (2). The thickening and narrowing of the lumen lead to complete occlusion and eventually graft failure.

Mutations in the proprotein convertase subtilisin/kexin type 9 (*Pcsk9*) gene were initially reported in patients with autosomal dominant hypercholesterolemia (4). Circulating PCSK9 binds to hepatic low-density lipoprotein receptor (LDLR) and mediates its degradation (5). Over-degradation of LDLR impairs cholesterol transport. Recently, the FOURIER and ODYSSEY clinical trials reported the therapeutic efforts of PCSK9 inhibitor (*i.e*., Evolocumab and alirocumab) on reducing the occurrence of cardiovascular events (6, 7). Although liver is the major source of PCSK9, it can also be secreted by extrahepatic organs (*e.g.*, intestine, neurons, and kidney), tissues (*e.g.*, adipose tissue), and cells (*e.g.*, vascular endothelial cells, macrophages, and vascular smooth muscle cells) (8, 9). Moreover, a recent investigation has suggested that PCSK9 plays an important role in atherosclerotic plaque development through a cholesterol-independent mechanism (10). PCSK9 is also involved in the pathogenesis of acute vascular injury (11), and the expression of PCSK9 is significantly increased in mouse aneurysms and human aortic dissection (12). Ding et al. have demonstrated that lipopolysaccharide increases the expression of PCSK9 in human endothelial cells and smooth muscle cells in a concentration-dependent manner (13, 14). Additionally, the combination of anti-programmed cell death protein 1 monoclonal antibody and Evolocumab has been shown to promote infiltration of cytotoxic T cells and inhibit tumor growth (15). In brief, these studies have shown that inhibition of PCSK9, a Federal Drug Administration-approved treatment for hyperlipidemia, reduces cardiovascular events, regulates inflammatory responses, and enhances the efficacy of immune checkpoint therapy in cancer treatment through a cholesterol-independent mechanism. However, the role of PCSK9 in GVD remains unclear.

The purpose of this study was to explore the role of PCSK9 in a mouse model of GVD. The serum level of PCSK9 in the allograft group was upregulated at 8 weeks after vascular transplantation compared to the isograft group. In addition, *Pcsk9* knockout (*Pcsk9^-/-^*) mice were tested for their development of graft vasculopathy compared to the wild-type (WT) mice. The pathological alterations, inflammatory cell infiltration, and proinflammatory cytokine expression in aortic grafts were detected, and the regulatory effects of PCSK9 on vascular smooth muscle cell (VSMC) migration and proliferation were examined. The effect of Evolocumab, a PCSK9 inhibitor, on GVD in humanized PCSK9 mice was also evaluated. The results of this study may be useful to find a treatment for GVD.

## Materials and Methods

### Mice

Male C57BL/6 (H-2b) and BALB/c (H-2d) mice (8–10 weeks old) were purchased from Charles River (Beijing, China). *Pcsk9^-/-^* mice (C57BL/6-Pcsk9^eml1Smoc^, stock no. NM-KO-200408) and hPCSK9 mice (C57BL/6-Pcsk9^em2/(hPCSK9)/Smoc^, stock no. NM-HU-00075) were generated by Shanghai Model Organisms (Shanghai, China). All animal experiments were approved by the Animal Care and Use Committee of Huazhong University of Science and Technology. All procedures were performed according to the NIH Guide for the Care and Use of Laboratory Animals.

### Abdominal Aortic Transplantation

Abdominal aortic transplantation was performed as previously described (16). For the allograft group, abdominal aortas of male BALB/c (H-2d) mice were orthotopically transplanted into fully major histocompatibility complex (MHC)-mismatched male C57BL/6 (H-2b) recipients, B6 background hPCSK9 or *Pcsk9^-/-^* recipients. For the isograft group, abdominal aortas of C57BL/6 (H-2b) mice were orthotopically transplanted into MHC-matched C57BL/6 (H-2b) recipients. To investigate the role of PCSK9 in GVD, Isograft group (WT B6 to WT B6), WT Allograft group (BALB/c to WT B6), and *Pcsk9^-/-^* Allograft group (BALB/c to *Pcsk9^-/-^* B6) were constructed. We also performed abdominal aortic transplantation on humanized PCSK9 mice to explore the role of PCSK9 inhibitors (Evolocumab) in GVD, and the following groups were included: Isograft group (hPCSK9 B6 to hPCSK9 B6 treatment with vehicle), IgG Group (BALB/c to hPCSK9 B6 treatment with isotype IgG), and Evolocumab group (BALB/c to hPCSK9 B6 treatment with Evolocumab).

### Enzyme-Linked Immunosorbent Assay

Serum samples were harvested from recipients at 8 weeks after surgery. The serum level of PCSK9 was measured by an ELISA kit according to the manufacturer’s protocol. The absorbance was measured at 450 nm. The concentration of PCSK9 was calculated based on the optical density value and the standard curve.

### Quantitative Real-Time Polymerase Chain Reaction

*qRT-PCR* was performed as previously described (17). Total RNA was isolated from graft vessels using Total RNA Extraction Kit (Solarbio, R1200, China) according to the manufacturer’s protocol. cDNA was synthesized by the cDNA synthesis kit (Abclonal, RK20400, China), according to the manufacturer’s instructions. Q-PCR were then performed using RealSYBR Mixture kit (CWBIO, CW0760M, China) according to the protocol. Gapdh was used as an internal control. The primer sequences are shown in **Table Sl**. Detailed information of critical commercial kits are showed in **Supplementary Table S2**.

### Tissue Histology and Morphometric Analysis

Graft vessels, liver, small intestine, and kidney were harvested at 2 or 8 weeks (time points have been explained in figure legends of each figure) after transplantation. And these samples were used for difference experiments, including Hematoxylin & eosin (H&E) staining, Masson’s staining, EVG staining, immunohistochemical staining, and immunofluorescent staining as previously described (18). ImageJ software was used to determine the intima-media (I/M) ratio, areas of intimal hyperplasia, areas of Masson’s staining and immunohistochemical quantitative analysis. During the quantitative analysis of collagen deposition and immunohistochemical, entire area has been measured.

### Western Blot

Western blot was performed as previously described (17). Total protein was extracted from cells or tissue samples using RIPA lysing buffer. The protein concentration of each sample was measured using the bicinchoninic acid protein assay kit. Western blot was performed according to a protocol of our laboratory. The membranes were separately incubated with the following primary antibodies. The antibodies used for Western blot are shown in **Table S2**.

### Isolation and Culture of Mouse Primary VSMCs and Bone Marrow-Derived Macrophages

VSMCs and BMDMs were cultured as previously described (19). Descending thoracic aortas were collected from mice at 8–10 weeks under sterile conditions. The aortas were then minced, and treated with 1% type I, II, IV collagenase (Worthington Biochemical Corporation) and 1% dispase for 1 h at 37°C. The cells were then harvested and resuspended in Dulbecco’s modified Eagle’s medium (DMEM) containing 20% fetal bovine serum (FBS) and 1% penicillin/streptomycin. Cells at passage 4–8 were used for the *in vitro* experiments. The purity of VSMCs was identified. Born marrow were extracted from the femurs and tibias of age-matched WT and *Pcsk9^-/-^* mice and then flushed with cold DMEM. After lysis of red blood cells, the BMDMs were harvested and resuspended in DMEM medium containing 10% FBS, 1% penicillin/streptomycin, and 20 ng/mL macrophage colony-stimulating factor (M-CSF; Peprotech, 315-02-10UG). The culture medium was replaced every other day with fresh medium containing 20 ng/mL M-CSF. After seven days of differentiation, the cells were counted and plated for further experiments.

### Transwell Assay

A transwell assay was performed to detect the migration capacity of VSMCs and BMDMs. Cells (three replicates per group) were added to the upper chamber (filled with serum-free medium) of a 24-well transwell plate (Corning 3422) at a density of 2 × 10^4^ cells/well. Serum samples were collected from allograft or isograft mice at 2 weeks after transplantation. A volume of 600 μL of culture medium supplemented with 10% serum was used for cell chemotaxis. After incubation for 48 h, the cells were fixed with 4% paraformaldehyde. Then, the cells on the upper side were wiped off. The invaded cells were stained with crystal violet (0.1%) solution for 10 min. Subsequently, the cells were visualized under a fluorescence microscope. Five random fields per slide were photographed. All experiments were performed at least three times, with each performed in duplicate.

### Cell Proliferation Assay

The proliferation rates of BMDMs and VSMCs were examined using a BeyoClick™ EdU Cell Proliferation Kit with Alexa Fluor 488 (Beyotime, C0071S), according to the manufacturer’s protocol. Briefly, serum was collected from allograft or isograft mice at 2 weeks after transplantation. Cells at a density of 5 × 10^4^ were plated in 24-well plates and incubated with 10% serum for 48 h. Then, the cells were incubated with 10 μM Edu for 6 h, fixed in 4% paraformaldehyde, and stained with Hoechst 33342. Finally, EdU-positive cells were counted by fluorescence microscopy.

### Statistical Analysis

Data are expressed as the mean ± SD (standard deviation). Unpaired t-test was used to compare two independent groups. One way ANOVA followed by Turkey’s post-hoc test or two-way ANOVA was used for comparisons among multiple groups. All statistical analyses were performed using SPSS 19.0 software. Plots were generated using GraphPad Prism 7.0a software (GraphPad Software, Inc., San Diego, CA) for Mac OS X. The significance level was determined at *p* < 0.05.

## Results

### PCSK9 is Upregulated in a Mouse Model of GVD

**Figure 1:**
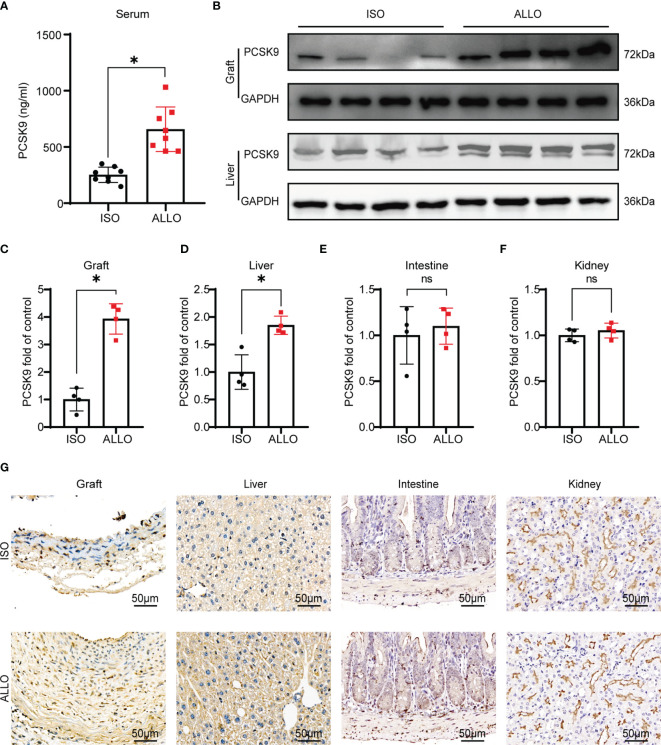
PCSK9 is increased in a mouse model of GVD. **(A)** The serum concentration of PCSK9 in isograft and allograft mice at 8 weeks after abdominal aortic transplantation was analyzed by ELISA (n = 8 per group). **(B)** PCSK9 expression in graft and liver of recipient mice was detected by Western blot. Samples were collected at 8 weeks after abdominal aortic transplantation. **(C–F)** The histogram shows the quantitative analysis of PCSK9 expression in the graft, liver, intestine, and kidney (n = 4, Isograft group was used as the control). **(G)** Immunohistochemical analysis of the graft, liver, intestine, and kidney from isograft group and allograft group (n = 4 per group, Scale bars: 50 μm). Bar graphs shown as mean ± SD. *p < 0.05; ns, not statistically significant. Unpaired Student’s *t*-test was used to compare two independent groups (ISO *vs.* ALLO). ISO, Isograft group; ALLO, Allograft group.

To explore whether PCSK9 is related to GVD, we measured the expression of PCSK9 in mice undergoing abdominal aorta transplantation. The abdominal aortas of BALB/c or C57BL/6 (B6) mice were orthotopically transplanted into B6 recipients. Serum samples and graft vessels were harvested at 8 weeks after surgery when allograft vasculopathy occurred. The allograft group showed a significantly higher level of PCSK9 in the serum compared to the isograft group (252.41 ± 68.32 ng/mL vs. 657.19 ± 198.34 ng/mL; *p* < 0.05). Similarly, in the graft vessels, the expression of PCSK9 in the allograft group was significantly greater than that of the isograft group (**Figure 1A**). PCSK9 is mainly secreted by the liver, but it is also expressed in extrahepatic tissues such as the intestine and kidney. Therefore, we further assessed the expression of PCSK9 in the liver, small intestine, and kidney of mice. The allograft group showed higher PCSK9 expression in the liver compared to the isograft group (**Figures 1B–D**), while no significant difference was observed in PCSK9 expression in the small intestine or kidney between the two groups (**Figure S1**; **Figures 1E, F**). The immunohistochemical staining results were consistent with those of western blot analysis (**Figure 1G**). These findings indicate that PCSK9 is involved in the pathogenesis of GVD.

### *Pcsk9* Depletion Ameliorates GVD in Mice

**Figure 2:**
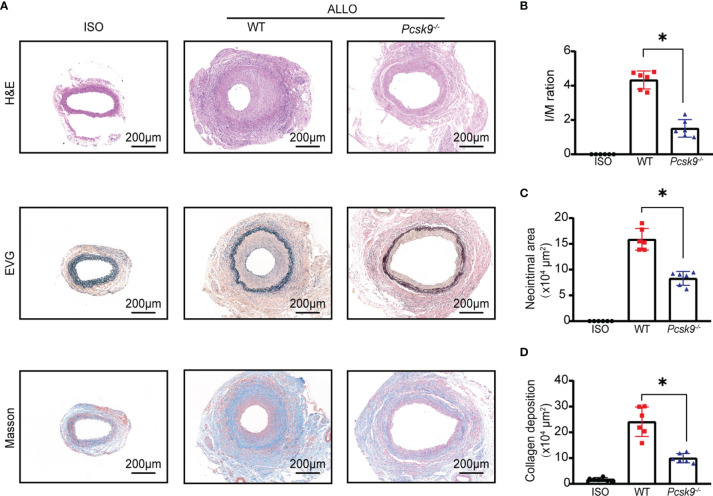
*Pcsk9* knockout ameliorates allograft vasculopathy in mice. **(A)** The sections of graft from the experimental group at 8 weeks after transplantation. Histogram analysis of the intima-media (I/M) ratio **(B)**, neointimal area **(C)**, and collagen deposition area **(D)**. (n = 6 per group. Scale bars: 200 μm). Bar graphs shown as mean ± SD. *p < 0.05. ISO, Isograft group; WT, allograft transplantation, recipients are WT mice; *Pcsk9^-/-^*: allograft transplantation, recipients are *Pcsk9^-/-^* mice. One way ANOVA followed by Turkey’s post-hoc test was used for comparisons among multiple groups.

To investigate the role of PCSK9 in GVD, we generated *Pcsk9^-/-^* mice. The abdominal aortas of BALB/c mice were orthotopically transplanted into *Pcsk9^-/-^* mice. Graft vessels were harvested at 8 weeks after surgery (**Figure 2A**). The histological analysis revealed that the *Pcsk9^-/-^* group had significantly less allograft vasculopathy (**Figure 2B**, I/M ratio: 4.34 ± 0.52 vs. 1.51 ± 0.50; *p* < 0.05) and a significantly smaller neointimal area (**Figure 2C**, 15.88 ± 2.09 × 10^4^ μm^2^ vs. 8.29 ± 1.35 × 10^4^ μm^2^; *p* < 0.05) compared with the WT group. Masson’s trichrome staining showed that collagen deposition in the allograft group was dramatically increased relative to the isograft group, whereas *Pcsk9* knockout significantly reduced the collagen deposition in the allograft mice (**Figure 2D**, 24.11 ± 5.72 × 10^4^ μm^2^ *vs.* 9.98 ± 1.82 × 10^4^ μm^2^; *p* < 0.05). Altogether, these data suggest that *Pcsk9* depletion attenuates allograft vasculopathy in a mouse model of GVD.

### *Pcsk9* Knockout Inhibits Macrophage Infiltration and Proinflammatory Cytokine Production

**Figure 3:**
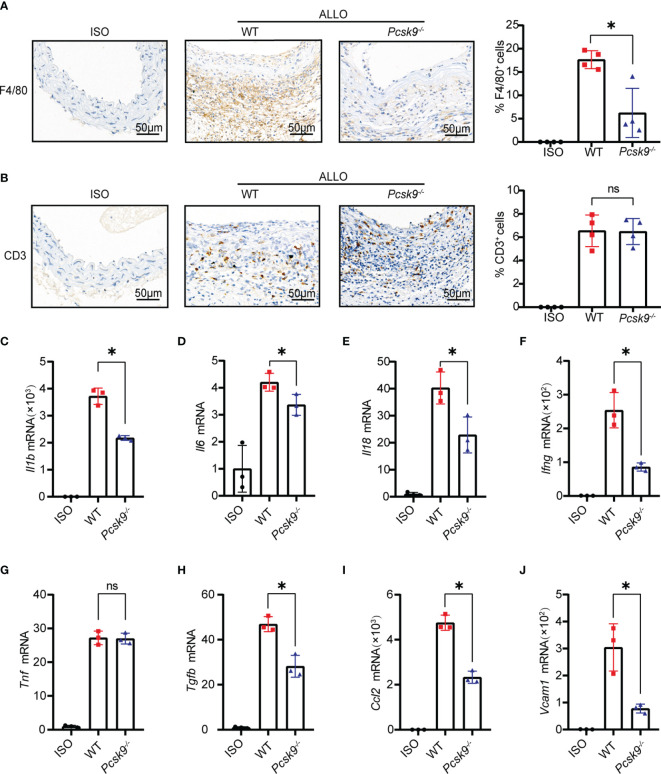
*Pcsk9* knockout inhibits macrophage infiltration and proinflammatory cytokine production. **(A)** Representative immunohistochemical staining results of F4/80^+^ macrophages. The histogram shows quantitative analysis of the percentages of F4/80^+^ macrophages per high-power field (n = 4). **(B)** Representative immunohistochemical staining results of CD3^+^ T cells. The histogram shows quantitative analysis of the percentages of CD3^+^ T cells per high-power field (n = 4). Graft used for immunohistochemical staining collected at 2 weeks after abdominal aortic transplantation. **(C7–J)** The mRNA levels of *Il1b*, *Il6*, *Il18*, *Ifng*, *Tnf*, *Tgfb*, *Ccl2*, and *Vcam1* in graft vessels were analyzed by qRT-PCR at 2 weeks after abdominal aortic transplantation (The isograft group was used as control. n = 3). One way ANOVA followed by Turkey’s post-hoc test was used for comparisons among multiple groups. Bar graphs shown as mean ± SD. *p < 0.05. ns, not statistically significant.

T cells and macrophages play a dominant role in GVD (20, 21). The infiltration of T cells and macrophages into the graft was detected by immunohistochemistry. *Pcsk9* knockout significantly reduced the infiltration of F4/80^+^ macrophages into the graft vessels (**Figure 3A**). However, there was no significant difference in CD3^+^ T cell infiltration between the *Pcsk9^-/-^* and WT groups (**Figure 3B**). We further analyzed the mRNA expression levels of proinflammatory cytokines, including *Il1b* (**Figure 3C**), *Il6* (**Figure 3D**), *Il18* (**Figure 3E**), *Ifng* (**Figure 3F**), *Tnf* (**Figure 3G**), *Tgfb* (**Figure 3H**), *Ccl2*, (**Figure 3I**), and *Vcam1* (**Figure 3J**), in graft vessels. *Il1b, Il6, Il18, Ifng, Tgfb, Ccl2*, and *Vcam1* were downregulated in the *Pcsk9^-/-^* group compared with the WT group. These findings indicate that *Pcsk9* knockout reduced the infiltration of macrophage and inhibited the expression of proinflammatory factors in graft vessels.

### *Pcsk9* Knockout Decreases the Migration and Proliferation of VSMCs

**Figure 4:**
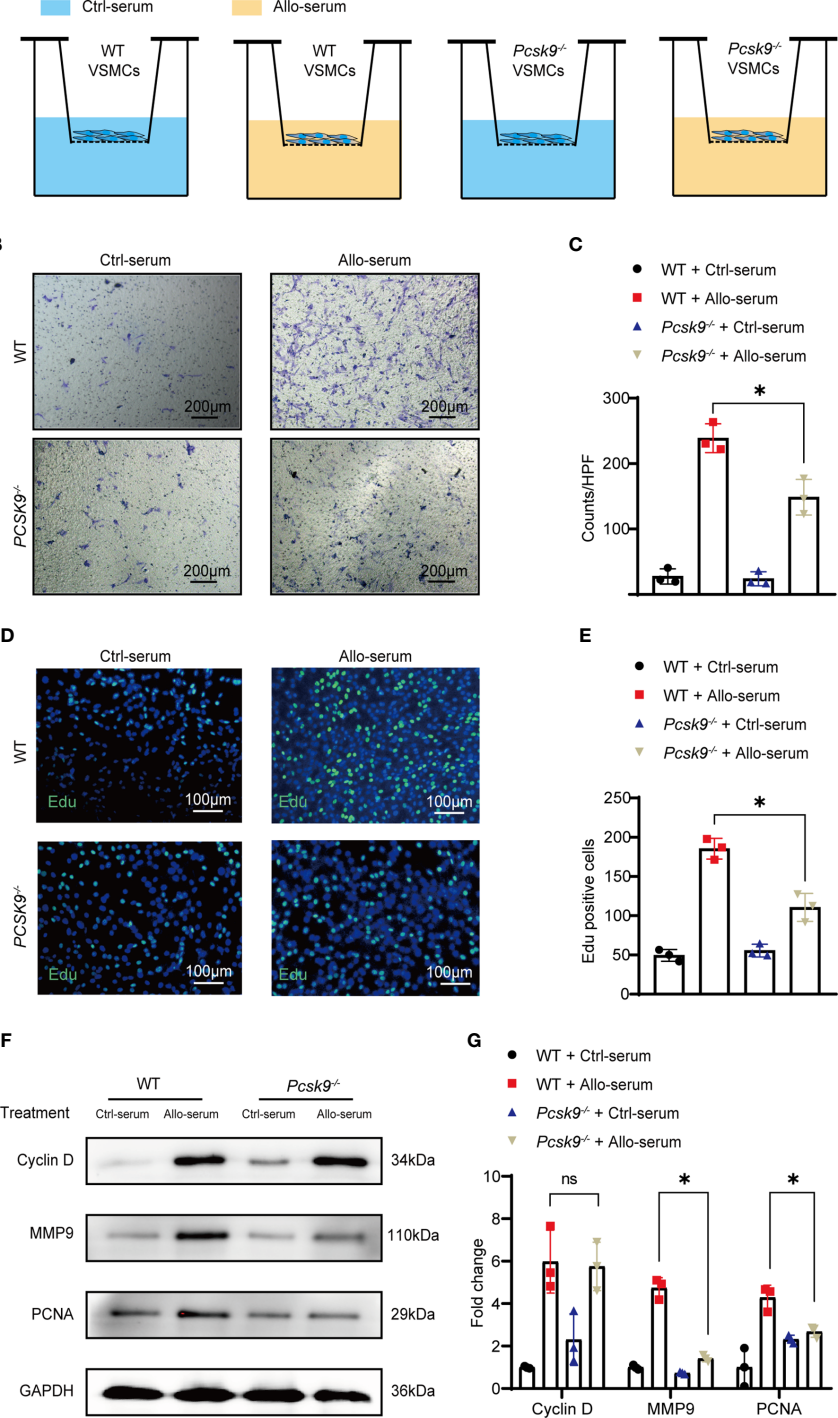
*Pcsk9* knockout decreases the migration and proliferation of VSMCs. **(A)** Schematic design of the VSMCs transwell assay. **(B)** VSMCs isolated from WT and *Pcsk9^-/-^* mice were seeded on the upper chamber of a transwell plate. Ctrl-serum or Allo-serum was used to stimulate cell migration to the bottom chamber for 48 h (scale bars: 200 μm). **(C)** The number of cells per high-power field was counted (n = 3). **(D)** The proliferative ability of VSMCs was detected by an EdU assay. VSMCs isolated from WT and *Pcsk9^-/-^* mice were stimulated with control or allogenic serum for 48 h (scale bars: 100 μm). **(E)** The number of EdU-positive cells per high-power field was counted (n = 3). **(F)** Western blot analysis of Cyclin D, MMP9, and PCNA expression in VSMCs isolated from WT and *Pcsk9^-/-^* mice after stimulation with control or allogenic serum for 24 h. **(G)** The histogram shows the quantitative analysis of Cyclin D, MMP9, and PCNA expression (n = 3, WT+ Ctrl-serum group was used as control). Two-way ANOVA and Simple effects tests were used for comparisons among multiple groups. Bar graphs shown as mean ± SD. *p < 0.05. ns, not statistically significant. Ctrl-serum, serum isolated from recipients received isograft transplantation; Allo-serum, serum isolated from recipients received allograft transplantation.

The migration and proliferation of VSMCs are key factors in allograft vasculopathy (22, 23). In addition, neointimal smooth muscle cells are mainly derived from the host (24). To explore the effect of PCSK9 on VSMCs migration and proliferation, we extracted VSMCs from WT and *Pcsk9^-/-^* mice. VSMCs were stimulated with the serum collected from recipient mice for 48 h. Transwell assay was performed to determine the migration capacity of VSMCs (**Figure 4A**). *Pcsk9* knockout significantly inhibited the migration of VSMCs (**Figures 4B, C**, 238.80 ± 21.94 vs. 148.60 ± 27.19; *p* < 0.05). Moreover, EdU staining showed that *Pcsk9* depletion inhibited the proliferation of VSMCs (**Figures 4D, E**, 185.40 ± 13.24 vs. 110.60 ± 17.89; *p* < 0.05). We also found that PCNA and MMP9 were downregulated in *Pcsk9*-knockout VSMCs (**Figures 4F, G**). However, *Pcsk9* knockout did not affect the migration or proliferation of macrophages compared with the control group (**Figure S2**). These results demonstrate that *Pcsk9* knockout inhibits the migration and proliferation of VSMCs stimulated with the serum collected from post-transplant mice.

### *Pcsk9* Knockout Represses NLRP3 Inflammasome Signaling in VSMCs

**Figure 5:**
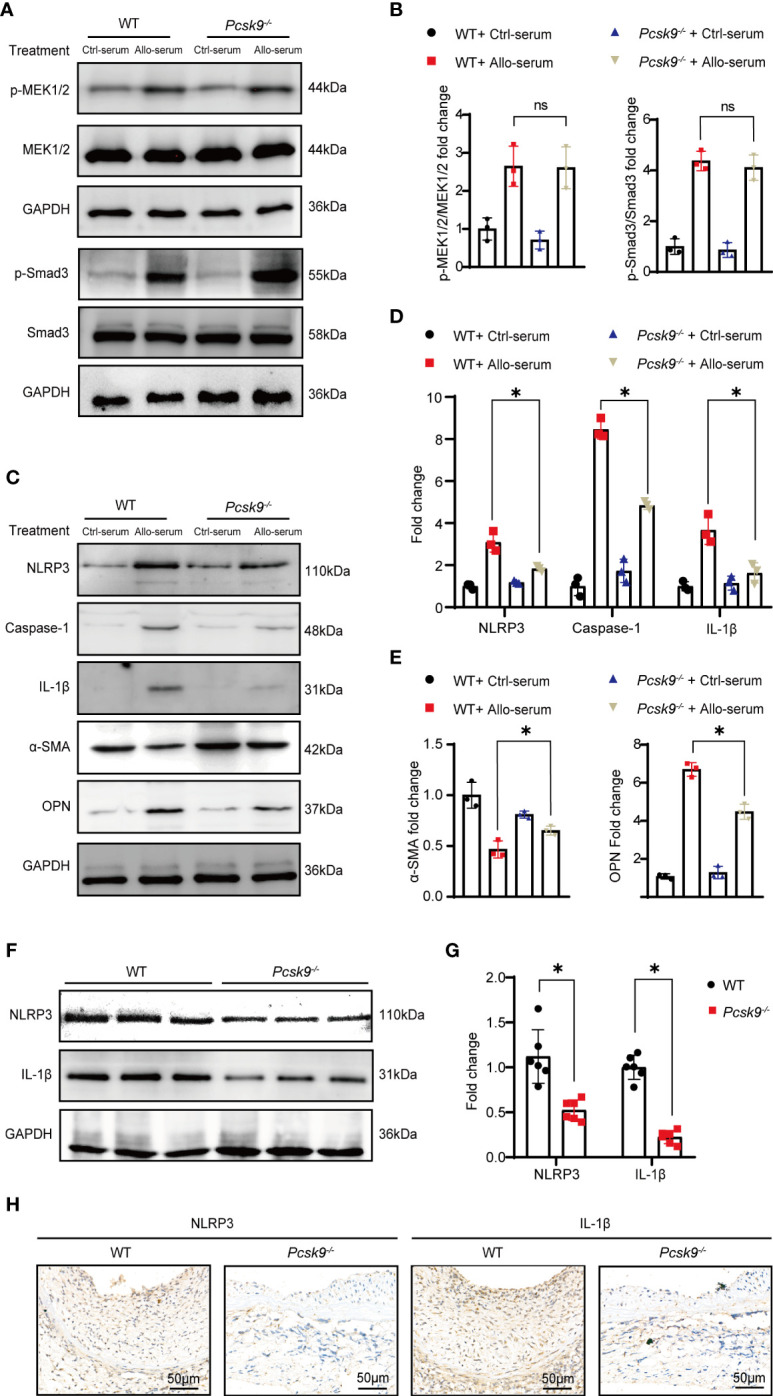
*Pcsk9* knockout represses NLRP3 inflammasome signaling in VSMCs. **(A)** Expression of p-MEK1/2 and p-Smad3 in VSMCs detected by Western blot. VSMCs were isolated from WT or *Pcsk9^-/-^* mice, and treated with Ctrl-serum or Allo-serum for 24 h. **(B)** The graphs show the quantitative analysis of p-MEK1/2 and p-Smad3 expression (n = 3, WT+ Ctrl-serum group was used as control). **(C)** Expression of NLRP3, Caspase-1, and IL-1β, α-SMA and OPN in VSMCs detected by Western blot. VSMCs were isolated from WT or *Pcsk9* ^-/-^ mice, and treated with Ctrl-serum or Allo-serum for 24 h. **(D)** The histogram shows the quantitative analysis of NLRP3, IL-1β, and Caspase-1expression (n = 3, WT+ Ctrl-serum group was used as control). **(E)** The histogram shows the quantitative analysis of α-SMA and OPN expression (n = 3, WT+ Ctrl-serum group was used as control). **(F)** Expression of NLRP3 and IL-1β in graft detected by western blot. Graft vessels were obtained at 8 weeks after abdominal aortic transplantation. **(G)** The graphs show the quantitative analysis of NLRP3 and IL-1β expression (n = 6, WT group was used as control). **(H)** Representative immunohistochemical staining results of NLRP3 and IL-1β in the graft vessels at 8 weeks after abdominal aortic transplantation (n = 6, Scale bars: 50 μm). Bar graphs shown as mean ± SD. *p < 0.05. ns, not statistically significant. Two-way ANOVA and Simple effects tests were used for comparisons among multiple groups **(B, D, E)**. Unpaired Student’s *t*-test was used to compare two independent groups **(G)**. Ctrl-serum, serum isolated from recipients received isograft transplantation; Allo-serum, serum isolated from recipients received allograft transplantation.

To further explore whether PCSK9 is involved in the signaling pathways related to proliferation and inflammation, we isolated VSMCs from WT and *Pcsk9^-/-^* mice and treated them with control or allograft serum for 24 h. Western blot analysis (**Figures 5A, B**) suggested that *Pcsk9* depletion did not affect the MAPK-MEK1/2 pathway and the TGF-β/Smad3 pathway. However, *Pcsk9* knockout inhibited the NLRP3 inflammasome pathway and phenotypic modulation of VSMCs (**Figures 5C–E**). Furthermore, graft vessels were harvested at 8 weeks after transplantation and analyzed by western blot and immunohistochemistry examination. The results showed that the expression levels of NLRP3 and IL-1β in the *Pcsk9^-/-^* group were lower than those in the WT group (**Figures 5F–H**). Collectively, these data imply that *Pcsk9* knockout inhibited the activation of NLRP3 inflammasomes in VSMCs.

### Evolocumab Attenuates GVD in Humanized *Pcsk9* Mice

**Figure 6:**
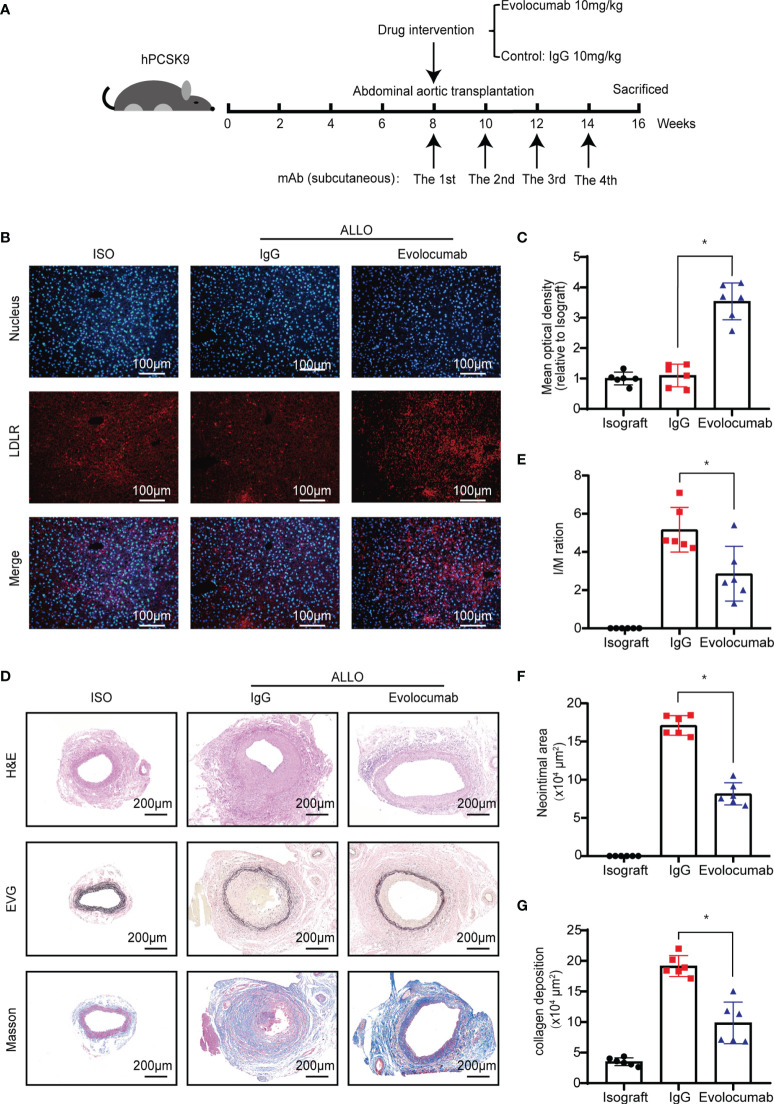
Evolocumab attenuates GVD in humanized PCSK9 mice. **(A)** Experimental design of Evolocumab intervention and tissue collection. **(B)** Immunofluorescence staining was performed to detect LDLR expression in the liver of recipient mice treated with different drugs (Scale bars: 100 μm). **(C)** The graphs show the quantitative immunofluorescence analysis of LDLR in the liver (n = 6, Isograft group was used as control). **(D)** H&E staining, Verhoeff’s Van Gieson, and Masson’s staining of graft vessels. Grafts were collected from recipient mice at 8 weeks after abdominal aortic transplantation (Scale bars: 200 μm). Histogram analysis of **(E)** the I/M ratio, **(F)** neointimal area, and **(G)** collagen deposition area (n = 6). One way ANOVA followed by Turkey’s post-hoc test was used for comparisons among multiple groups. Bar graphs shown as mean ± SD. *p < 0.05. ISO, Isograft group; IgG, allograft transplantation recipients treated with IgG; Evolocumab, allograft transplantation recipients treated with Evolocumab.

Evolocumab, a neutralizing antibody to human PCSK9, has been tested in clinical trials for familial hypercholesterolemia or hyperlipidemia that cannot be controlled by statins (25). To investigate whether Evolocumab would be an effective treatment for GVD, we performed allogeneic abdominal aorta transplantation surgery, during which the abdominal aortas of BALB/c mice were orthotopically transplanted into B6 background humanized PCSK9 mice (hPCSK9) recipients. After transplantation, the recipients were subcutaneously injected with Evolocumab (10 mg/kg) or IgG (10 mg/kg) every 2 weeks for 8 weeks (**Figure 6A**). Western blot (**Figures S3A, B**) and immunofluorescence analyses (**Figures 6B, C**) showed that LDLR was significantly upregulated in the Evolocumab-treated group at 8 weeks after transplantation. Graft vessels were collected for histological staining (**Figure 6D**), and the histological analyses showed that Evolocumab-treated mice had significantly reduced vascular stenosis (**Figure 6E**, I/M ratio: 5.16 ± 1.16 vs. 2.86 ± 1.43; *p* < 0.05) and a significantly smaller area of intimal hyperplasia (**Figure 6F**, 17.10 ± 1.28 × 10^4^ μm^2^ vs. 8.15 ± 1.46 × 10^4^ μm^2^; *p* < 0.05) compared with the IgG-treated group. Masson’s trichrome staining showed that Evolocumab treatment significantly decreased collagen deposition in the hPCSK9 mice (**Figure 6G**, 19.16 ± 1.74 × 10^4^ μm^2^ vs. 9.86 ± 3.39 × 10^4^ μm^2^; *p* < 0.05). Taken together, Evolocumab inhibited the progression of GVD in hPCSK9 mice.

## Discussion

GVD remains the Achilles’ heel of long-term survival after solid-organ transplantation, including cardiac allograft vasculopathy after heart transplantation. Multiple factors have been shown to contribute to the development of GVD, such as hyperlipidemia, ischemia-reperfusion injury, and immunosuppressive pharmacotherapy (26–28). Clinical studies have also demonstrated that statins and mTOR inhibitors can protect GVD and have been effectively used in clinical practice (29, 30). However, more effective and specific targets are still urgently needed. The mouse model of aortic transplantation has been widely used to simulate vascular pathology in GVD (31), mainly in cardiac allograft vasculopathy (32, 33). As accelerated transplant vasculopathy and chronic allograft nephropathy are the pathological features of chronic kidney allograft rejection, this model could potentially be used to mimic transplant vasculopathy in chronic kidney allograft rejection. In this study, we found that PCSK9 was upregulated in the serum, graft vessels, and liver of mice with GVD. Morphologically, *Pcsk9* knockout significantly reduced vascular stenosis, the area of intimal hyperplasia, and collagen deposition in the mouse model of GVD. Depletion of the *Pcsk9* gene also inhibited macrophage recruitment and the mRNA expression of proinflammatory cytokines in graft vessels. We further showed that *Pcsk9* knockout reduced the migration and proliferation of VSMCs *in vitro*. Meanwhile, *Pcsk9* knockout inhibited activation of the NLRP3 inflammasome signaling pathway in VSMCs both *in vivo* and *in vitro*. Additionally, Evolocumab significantly ameliorated the progression of GVD in hPCSK9-recipient mice.

It has been shown that PCSK9 is upregulated in VSMCs stimulated with lipopolysaccharide, which is important in inflammation (34). Here, we simulated GVD-induced vascular stenosis by establishing a mouse model of abdominal aortic transplantation and found that PCSK9 was highly expressed not only in the serum and liver of allogenic mice but also in the lesion site of the graft. Furthermore, *Pcsk9* knockout significantly ameliorated graft vascular disease in mice. These results suggest the involvement of PCSK9 in the process of graft vascular vasculopathy. As both immune and nonimmune pathways contribute to vascular injury in solid-organ transplants, macrophages and T lymphocytes are implicated in the chronic rejection of organ transplantation (21, 35). Cytokines, including proinflammatory molecules, cell adhesion molecules, and chemokines, are essential for the recruitment of inflammatory cells and phenotypic transformation of smooth muscle cells (36). In this study, *Pcsk9* depletion significantly inhibited macrophage recruitment and proinflammatory cytokine production.

Vascular graft stenosis refers to the migration of receptor-derived smooth muscle-like cells to the lesion site and subsequent hyperproliferation (24). In this study, we showed that *Pcsk9* knockout inhibited the proliferation of VSMCs as measured by the EdU assay, which was related to the downregulation of PCNA. *Pcsk9* knockout might also inhibit the migration of VSMCs, and the transwell result is influenced by various factors, not only the cell migration ability, but also cell viability (proliferation, apoptosis, necrosis). PCSK9 is also expressed in macrophages. Badimon et al. have demonstrated that PCSK9 upregulates TLR4/NF-κB, favors inflammation, and participates in lipid uptake in human macrophages (37). However, we found that *Pcsk9* knockout did not affect the migration and proliferation of BMDMs. The differences in these results may be due to the different sources of macrophages. The MAPK-MEK1/2 pathway has been shown to regulate the proliferation of smooth muscle cells and tumor cells (38, 39). Furthermore, Li et al. have confirmed that the MAPK-MEK1/2 pathway is highly expressed in graft vasculopathy and that MEK1/2 inhibitors can alleviate graft vascular stenosis (40). Our previous study also has shown that the TGF-β/Smad3 pathway regulates the proliferation of smooth muscle-like cells and promotes the production of extracellular matrix (17). Here, we demonstrated that PSCK9 depletion in VSMCs did not affect these pathways. However, a number of studies have reported an interaction between PCSK9 and NLRP3. Ding et al. have found that NLRP3 inflammasomes regulate the secretion of PCSK9 in peritoneal macrophages (41). In addition, the knockout of *Pcsk9* in cardiomyocytes has been demonstrated to inhibit NLRP3 inflammatory signaling (NLRP3, ASC, caspase-1, IL-1β, and IL-18) and chronic myocardial ischemia (42). Previous studies also have shown that NLRP3 inflammasomes play an important role in migration, proliferation, phenotypic transformation, and other inflammatory responses of VSMCs (41, 43, 44). In this study, we showed that *Pcsk9* knockout inhibited the expression levels of NLRP3, caspase-1, and IL-1β in VSMCs. Mature VSMCs are quiescent and express contractile genes (e.g., a-SMA). They transform to proinflammatory phenotype (e.g., OPN) in response to vascular injury (45). *Pcsk9* knockout increased the a-SMA expression and repressed the OPN expression in VSMCs treated with allograft serum. These results indicated that *Pcsk9* knockout might promote contractile phenotype but repress proinflammatory phenotype of VSMCs. Meanwhile, NLRP3 and IL-1β were also downregulated in the neointimal grafts of *Pcsk9^-/-^* recipient mice. These results suggest that PCSK9 could regulate inflammatory responses in VSMCs probably through NLRP3 inflammasomes. However, Guo, Y., et al. proved that PCSK9 inhibited proliferation, and ultimately leaded to vascular stiffness in arterial ligation mouse model. As GVD GVD is associated with both immune responses and nonimmune factors, which dramatically different from the pathological process of arterial ligation induced arterial stiffness. In addition, PCSK9 is a protein with multiple biological effects (46–48). Therefore, PCSK9 may exert different effects on VSMCs in the different animal models.

Evolocumab (a PCSK9 inhibitor) is a novel lipid-lowering drug that has been shown to decrease the incidence of myocardial infarction and stroke (49). In addition, Evolocumab has also been used in heart transplantation recipient on a small scale (25, 50, 51). Here, we found that Evolocumab, similar with *Pcsk9* knockout, reduced allograft vasculopathy in hPCSK9 mice. The preventive effect of Evolocumab on cardiac allograft vasculopathy in heart transplant recipients is also being investigated (Clinical Trials. gov Identifier: NCT03734211). These studies focused on the lipid-lowering function of Evolocumab. However, the roles of PCSK9 in immune regulation and inflammatory response might also contribute to the development of GVD after organ-transplantation. Our results desmonstrated that PCSK9 participated in the development of GVD through a cholesterol-independent mechanism. However, larger-scale clinical trials are needed to evaluate the potential clinical use of Evolocumab for the treatment of GVD. In addition, the protective effect of Pcsk9 knockout or Evolocumab in GVD model may be related with the lipid-lowering function. There are some limitations in this current study. This study is exclusively performed in mice and with murine cells, and human data is lacked, as the integration of human data and murine data can improve the level of study and add a more translational aspect of the findings. Then, whether Evolocumab affect the recruitment of macrophages and the changes of inflammatory factors after transplantation has not been evaluated in our study.

In conclusion, this study confirms the upregulation of PCSK9 in a mouse model of GVD. In addition, *Pcsk9* knockout inhibits NLRP3 inflammasome signaling in VSMCs and reduces allograft vasculopathy. These findings suggest that PCSK9 is a promising target for the treatment of GVD.

## Data Availability Statement

The original contributions presented in the study are included in the article/**Supplementary Material**. Further inquiries can be directed to the corresponding authors.

## Ethics Statement

The animal study was reviewed and approved by Animal Care and Use Committee of Huazhong University of Science and Technology.

## Author Contributions

YZ, XZ and ZC conceived and designed the experiments. JX, JW and CZ drafted the manuscript. YZ and HX performed the experiments. JY established the animal models. JC, YL and YN analyzed the data. All authors contributed to the article and approved the submitted version.

## Funding

This work received financial support from the National Natural Science Foundation of China (82071803,81730015), Natural science fund of Hubei Province (2019AAA032) and Fundamental Research Funds for the Central Universities (2021GCRC037).

## Conflict of Interest

The authors declare that the research was conducted in the absence of any commercial or financial relationships that could be construed as a potential conflict of interest.

## Publisher’s Note

All claims expressed in this article are solely those of the authors and do not necessarily represent those of their affiliated organizations, or those of the publisher, the editors and the reviewers. Any product that may be evaluated in this article, or claim that may be made by its manufacturer, is not guaranteed or endorsed by the publisher.

## Abbreviations

BMDMs, Bone marrow-derived macrophages; Ccl2, C-C chemokine ligand 2; ELISA, Enzyme-linked immunosorbent assay; EVG, Verhoeff’s Van Gieson; GVD, Graft vascular disease; HE, Hematoxylin-eosin; I/M, Intima-media ratio; Ifng, Interferon gamma; IL-18, Interleukin 18; IL-6, Interleukin 6; LDLR, Low density lipoprotein receptor; mRNA, Messenger RNA; OPN, Osteopontin; PCNA, Proliferating Cell Nuclear Antigen; PCSK9, Proprotein convertase subtilisin/kexin type 9; qRT-PCR, Quantitative real-time polymerase chain reaction; RT-PCR, Real-time-quantitative polymerase chain reaction; Tgfb, Transforming growth factor-beta; Tnf, Tumor necrosis factor; Vcam1, Vascular cell adhesion molecule 1; VSMC, Vascular smooth muscle cell; WT, Wild-type; α-SMA, α-smooth muscle actin; PNCA, Proliferating cell nuclear antigen; mTOR, Molecular Target of Rapamycin.
