# Supplementary figures and images for: Targeting PCSK9 Ameliorates Graft Vascular Disease in Mice by Inhibiting NLRP3 Inflammasome Activation in Vascular Smooth Muscle Cells
Source: Front Immunol. 2022 May 26;13:894789. doi: 10.3389/fimmu.2022.894789 (PMC9204514; doi:10.3389/fimmu.2022.894789)

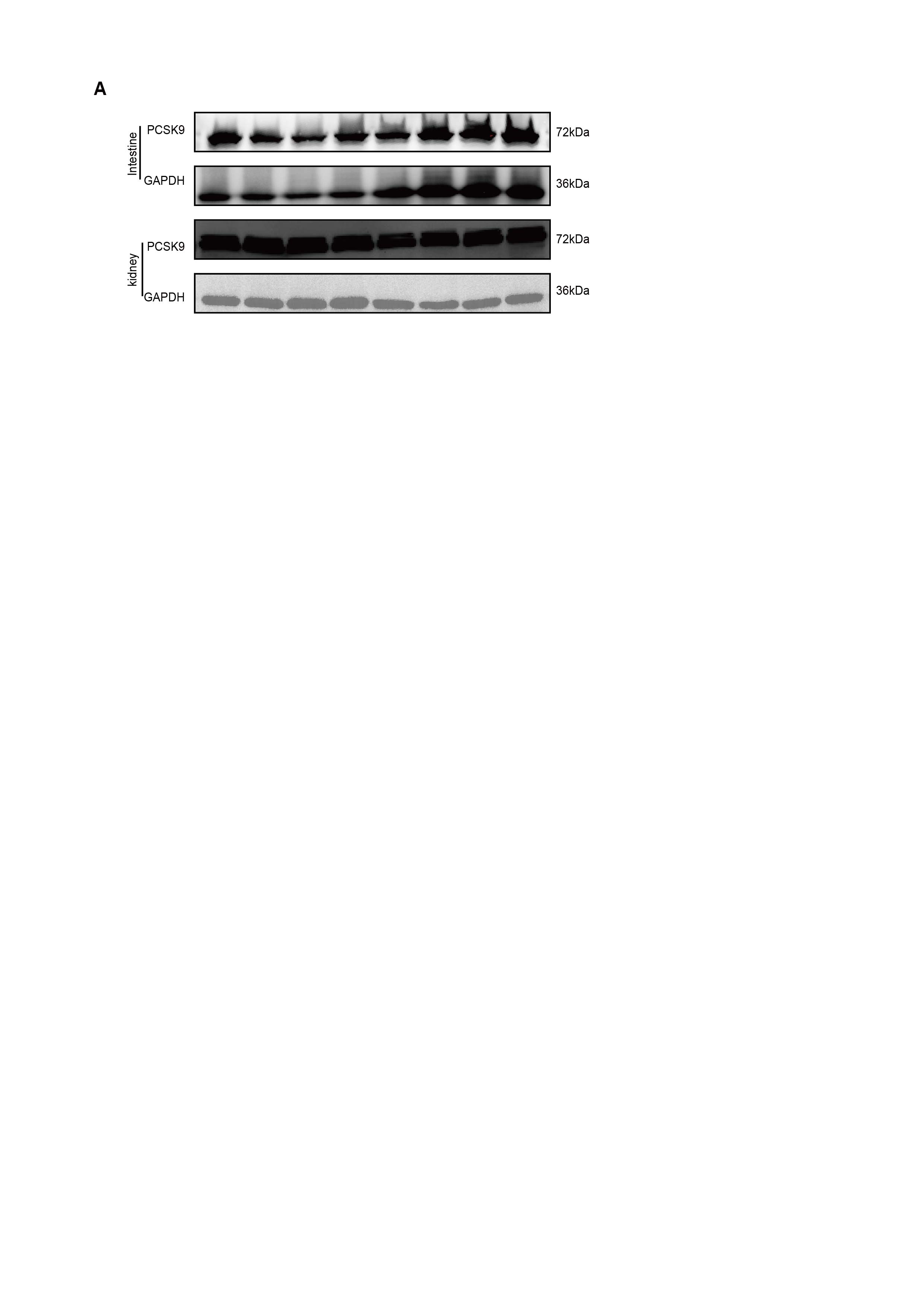

Supplement: Supplementary file 1 [file Image_1.jpeg]

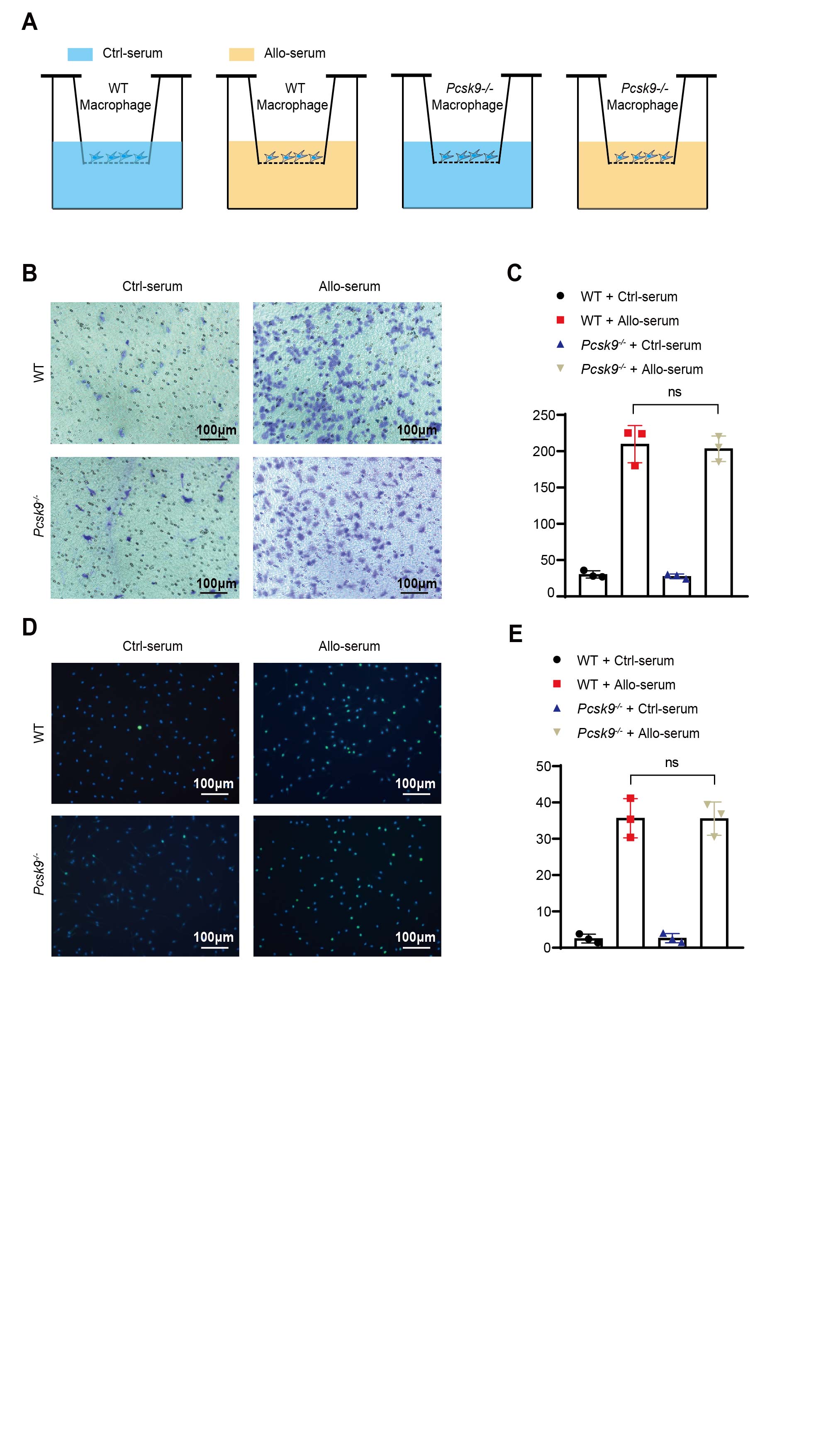

Supplement: Supplementary file 2 [file Image_2.jpeg]

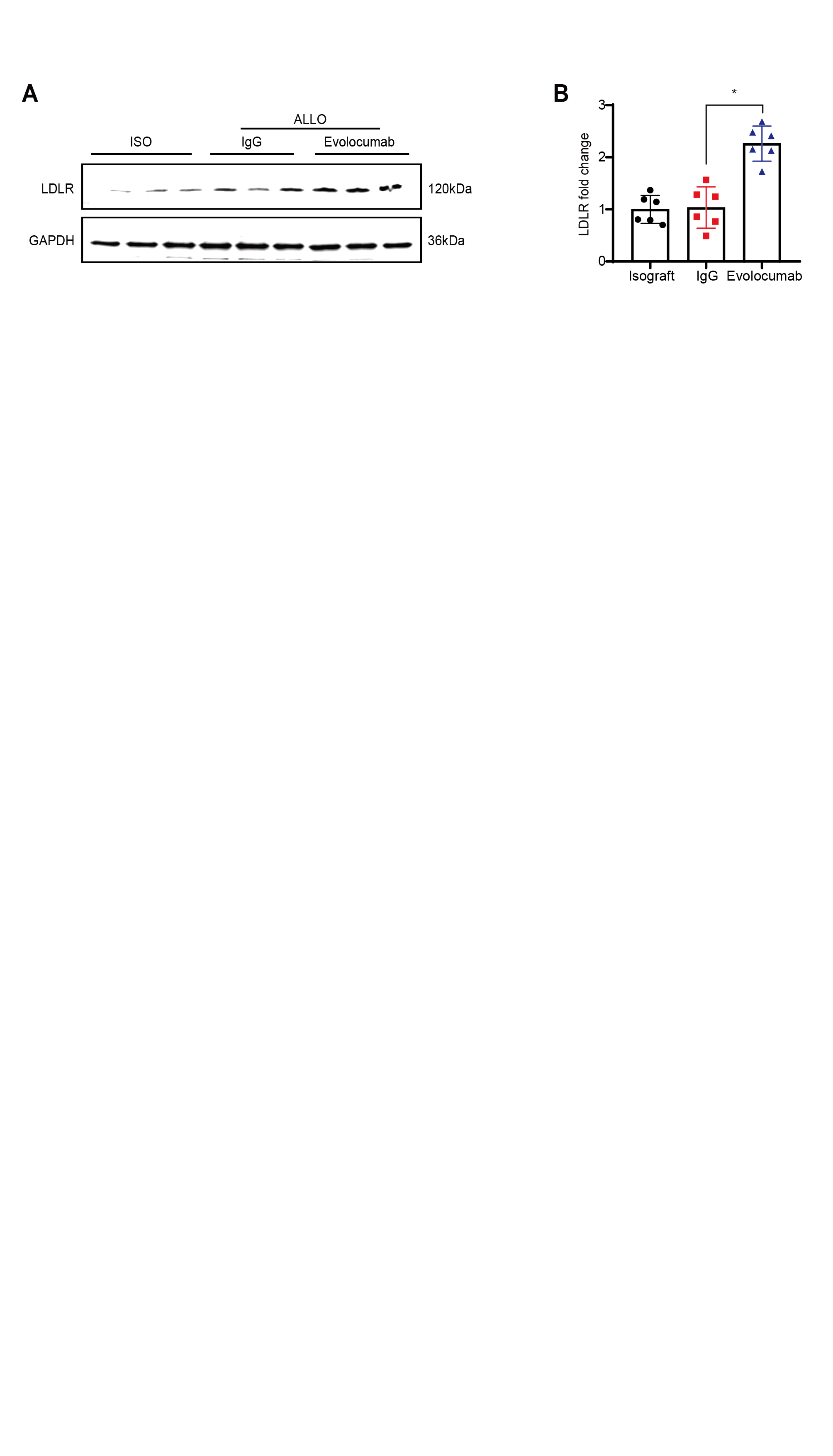

Supplement: Supplementary file 3 [file Image_3.jpeg]
