# Supplementary material for: Targeting PCSK9 Ameliorates Graft Vascular Disease in Mice by Inhibiting NLRP3 Inflammasome Activation in Vascular Smooth Muscle Cells
Source: Front Immunol. 2022 May 26;13:894789. doi: 10.3389/fimmu.2022.894789 (PMC9204514; doi:10.3389/fimmu.2022.894789)
Supplement: Supplementary file 4 [file Table_1.docx]

**Table S1. Detail information of primer pairs used in qRT-PCR.**

| **Gene** Sequence (5’-3’) |
| --- |
| *Il1* Forward 5′-GCAACTGTTCCTGAACTCAACT-3′  Reverse 5′-ATCTTTTGGGGTCCGTCAACT-3′  ***Il6*** Forward 5′-TAGTCCTTCCTACCCCAATTTCC-3′  Reverse 5′-TTGGTCCTTAGCCACTCCTTC-3′  ***Il18*** Forward 5′-ACTGTACAACCGCAGTAATACGC-3′  Reverse 5′-AGTGAACATTACAGATTTATCCC-3′  ***Ifng*** Forward 5′-ATGAACGCTACACACTGCATC-3′  Reverse 5′- CCATCCTTTTGCCAGTTCCTC-3′  ***Tnf*** Forward 5′-CCCTCACACTCAGATCATCTTCT-3′  Reverse 5′-GCTACGACGTGGGCTACAG-3′  ***Tgfb*** Forward 5′-TACCATGCCAACTTCTGTCTGGGA-3′  Reverse 5′-ATGTTGGACAACTGCTCCACCTTG-3′  ***Ccl2*** Forward 5′-TTAAAAACCTGGATCGGAACCAA-3′  Reverse 5′-GCATTAGCTTCAGATTTACGGGT-3′  ***Vcam1*** Forward 5′-CCGGCATATACGAGTGTGAA-3′  Reverse 5′-GATGCGCAGTAGAGTGCAAG-3′  ***Gapdh*** Forward 5′-AGGTCGGTGTGAACGGATTTG-3′  Reverse 5′-TGTAGACCATGTAGTTGAGGTCA-3′ |
