# Supplementary material for: Targeting PCSK9 Ameliorates Graft Vascular Disease in Mice by Inhibiting NLRP3 Inflammasome Activation in Vascular Smooth Muscle Cells
Source: Front Immunol. 2022 May 26;13:894789. doi: 10.3389/fimmu.2022.894789 (PMC9204514; doi:10.3389/fimmu.2022.894789)
Supplement: Supplementary file 5 [file Table_2.docx]

**Table S2 Key resources table**

| Reagent or resources | Source | Identifier |
| --- | --- | --- |
| Antibodies |  |  |
| Anti-PCSK9 | Thermofisher (USA) | PA5-96836 |
| Anti-Cyclin D | ABclonal (China) | A10757 |
| Anti-MMP9 | Abclonal (China) | A2095 |
| Anti-PCNA | Bioworld (China) | MB9012 |
| Anti-Osteopontin | Beyotime (China) | AF7665 |
| Anti-Caspase-1 | Beyotime (China) | AF1681 |
| Anti-NLRP3 | Signalway antibody (USA) | 49012 |
| Anti-MEK1/2 | Thermofisher (USA) | MA5-31998 |
| Anti-SMAD3 | Signalway antibody (USA) | 48783 |
| Anti-Phospho-Smad3 (Ser423/425) | HUABIO (USA) | ET1609-41 |
| Anti-IL lβ | Abclonal (China) | A1112 |
| Anti-Phospho-MAP2K1/2  (Ser217/221) | Abclonal (China) | AP0209 |
| Anti-LDLR | Proteintech (USA) | 10785-1-AP |
| Anti- Smooth Muscle Actin | Proteintech (USA) | 55135-1-AP |
| HRP-Conjugated GAPDH Monoclonal Antibody | Proteintech (USA) | HRP-60004 |
| Anti-CD3 | Servicebio (China) | GB13022-3 |
| Anti- F4/80 | Servicebio (China) | GB11027 |
| IgG (H+L) Cross-Adsorbed Goat anti-Rat, Alexa Fluor™ 647 | Invitrogen (USA) | A21247 |
|  |  |  |
| Critical Commercial Assays |  |  |
| Mouse PCSK9 ELASA kit | Assay Genie (Ireland) | MOFI00083 |
| ABScript II cDNA Fist-Strand Synthesis Kit | Abclonal (China) | RK20400 |
| RealSYBR Mixture | CWBIO (China) | CW0760M |
| Total RNA Extraction Kit | Solarbio (China) | R1200 |
| BeyoClick^TM^ EdU Cell Proliferation Kit | Beyotime (China) | C0071S |
| Pierce™ BCA Protein Assay Kit | Thermofisher (USA) | 23225 |
| Transwell® Permeable Supports, Polycarbonate (PC) Membrane | Corning (USA) | 3422 |
| Chemicals, Drugs and  Recombinant Proteins |  |  |
| RIPA Lysis Buffer | Sangon Biotech (China) | C500005 |
| Protease Inhibitor Cocktail | Sangon Biotech (China) | C600387 |
| Total RNA Extractor (Trizol) | Sangon Biotech (China) | B511311 |
| Recombinant  Murine M-CSF | PeproTech (USA) | 315-02-10UG |
| InVivoMAb human IgG2 isotype control | BioXcell (USA) | BE0301 |
| Evolocumab Injection | Amgen (USA) | S20180021 |
